# A document-level information extraction pipeline for layered cathode materials for sodium-ion batteries

**DOI:** 10.1038/s41597-024-03196-1

**Published:** 2024-04-11

**Authors:** Yuxiao Gou, Yiping Zhang, Jian Zhu, Yidan Shu

**Affiliations:** 1 School of Materials Science and Engineering, Sun Yat-sen University, Guangdong, China; 2 The Key Laboratory of Low-carbon Chemistry & Energy Conservation of Guangdong Province, Guangdong, China

**Keywords:** Batteries, Information technology

## Abstract

Natural language processing techniques enable extraction of valuable information from large amounts of published literature for the application of data science and technology, i.e. machine learning in the field of materials science. Nevertheless, the automated extraction of data from full-text documents remains a complex task. We propose a document-level natural language processing pipeline for literature extraction of comprehensive information on layered cathode materials for sodium-ion batteries. The pipeline enhances entity recognition with contextual supplementary information while capturing the article structure. Finally, a heuristic multi-level relationship extraction algorithm is employed in relation extraction to extract experimental parameters and complex performance relationships respectively. We successfully extracted a comprehensive dataset containing 5265 records from 1747 documents, encompassing essential information such as chemical composition, synthesis parameters, and electrochemical properties. By implementing our pipeline, we have made significant progress in overcoming the challenges associated with data scarcity in battery informatics. The extracted datasets provide a valuable resource for further research and development in the field of layered cathode materials.

## Introduction

Sodium-ion batteries (SIBs) have garnered significant attention due to their similar working principles to lithium-ion batteries and the abundant, cheap, and widely distributed sodium resources. In the field of SIBs, various types of materials, including transition metal oxides^1,2^, Prussian blue compounds^3^, polyanion-type compounds^4^, have been studied as cathode materials for sodium-ion batteries. Among these, layered transition metal oxides (LTMOs) including NaxTMO2 (TM = Fe, Ni, Co, Mn, etc.) have become the most promising cathode material for SIBs owing to its large specific capacity, high ionic conductivity, and feasible preparation conditions. This has led to extensive research efforts aimed at developing SIBs as a viable alternative to lithium-ion batteries for large-scale energy storage systems.

In recent years, the academic community has witnessed a significant increase in the number of published research papers specifically dedicated to investigating LTMOs as cathode materials for SIBs. However, many of these papers still rely on traditional trial-and-error methods for material development, which can be time-consuming. One potential solution to accelerate the design and development of new materials is to leverage data science techniques and adopt a systematic “materials-by-design” approach. By utilizing data science techniques, researchers can analyze large amounts of data and extract valuable insights to guide the design of new materials. This approach has the potential to significantly speed up the discovery of new cathode materials for SIBs. However, the non-machine-readable formats of publication leads to data scarcity in the field of battery materials informatics. This scarcity hinders the training of property predictors, as it requires laborious manual curation of relevant data from the literature. Ling^5^ provided a comprehensive summary of the various types of datasets that are applicable for battery informatics studies. However, it is noteworthy that there is still a lack of datasets specifically dedicated to the investigation of the cycling and rate performance of layered cathode materials for SIBs. Therefore, developing methods for automatically extracting data rapidly and accurately has increasingly become a necessity.

The application of natural language processing (NLP) techniques to automatically extract data on organic and inorganic chemical substances from articles in the fields of chemistry and materials science has shown promising results^6–9^. Information extraction (IE) from written text is an extensively studied area within NLP. The idea of “self-supervised learning” through transformer-based models such as Bidirectional Encoder Representations from Transformers (BERT)^10^, SciBERT^11^ and BatteryBERT^12^, which are pre-trained on massive corpora of unlabeled text to learn contextual embeddings and further fine-tuning through supervised learning on specific datasets, enabling them to surpass the performance of BERT in context-aware NLP applications such as text classification, entity recognition (NER), and question answering^13^, is the dominant paradigm of information extraction today. A common workflow for text classification, NER and relation extraction is to feed labelled inputs to BERT and use the output vector embedding of each sentence or word along with the corresponding labels as inputs to a task-specific machine learning model that learns to predict these labels. In practice, due to constraints such as the size of the labelled data and the complexity of the task, a mixture of model and rule processing is adopted for a given task to achieve a balance between accuracy and computational efficiency^14^. For example, the latest version of ChemDataExtractor version 2.1, incorporates a NER system that combines Bert, Conditional Random Field (CRF), rules, and dictionaries. This combined approach has shown impressive performance, achieving results that are comparable to the state-of-the-art for recognizing both organic and inorganic entities^15^.

On studies pertaining to batteries, El-Bousiydy^16^ *et al*. extracted ten characteristics related to the electrochemical performance of lithium-ion batteries (LIBs) (mass loading, porosity, thickness, surface area, electrode composition, electrolyte volume, electrolyte composition, separator, voltage cut-off, and voltage range) and provided a general perspective. Kononova^17^ *et al*. used an automated extraction pipeline to extract data on inorganic chemical substances containing cathode materials for LIBs only from solid-state synthesis passages of scientific publications to create a solid-state synthesis “codified recipes” database. Huang^18^ *et al*. modified ChemDataExtractor to automatically generate information from more than 229,000 papers related to battery. A large database including five material properties: capacity, voltage, conductivity, Coulombic efficiency, and energy was constructed, where rate capacity and cycling capacity are not separated, leading to the main problem that currents in the form of “C-rates” cannot be standardized. In summary, the majority of previous work utilizing NLP techniques in the field of materials science focused on constructing individual databases on either material synthesis parameters or properties through extracting from abstracts or partial texts. However, to the best of our knowledge, limited efforts have been made to comprehensively extract synthesis parameters and performance data from full-text and build a systematic database covering the information on both synthesis and performance of materials, specifically for battery cathode materials.

In this work, we construct a generic pipeline for extracting both material properties and synthetic parameter data of cathode materials of SIB. To the best of our knowledge, this work represents the first comprehensive extraction method of synthesis parameters and properties for sodium layered cathode materials generated based on literature data. Each instance in the dataset not only covers the chemical composition and synthesis parameters, such as sintering temperature, sintering time, but also functional properties such as cycling capacity, number of cycles, rate capacity, test current, and test voltage range. In total, 1747 articles on sodium cathode materials were filtered (See **document classification** for detailed filtering strategies) from a corpus of 63447 articles from Elsevier and Royal Society of Chemistry (RSC) publishers and a dataset of 5265 instances were extracted automatically. For extraction of complex relation, including materials, synthesis parameters, cycling and rate performance, our method presents a composite F1 score of 81.14% and 82.67% from articles from Elsevier and RSC respectively.

## Results

**Fig. 1:**
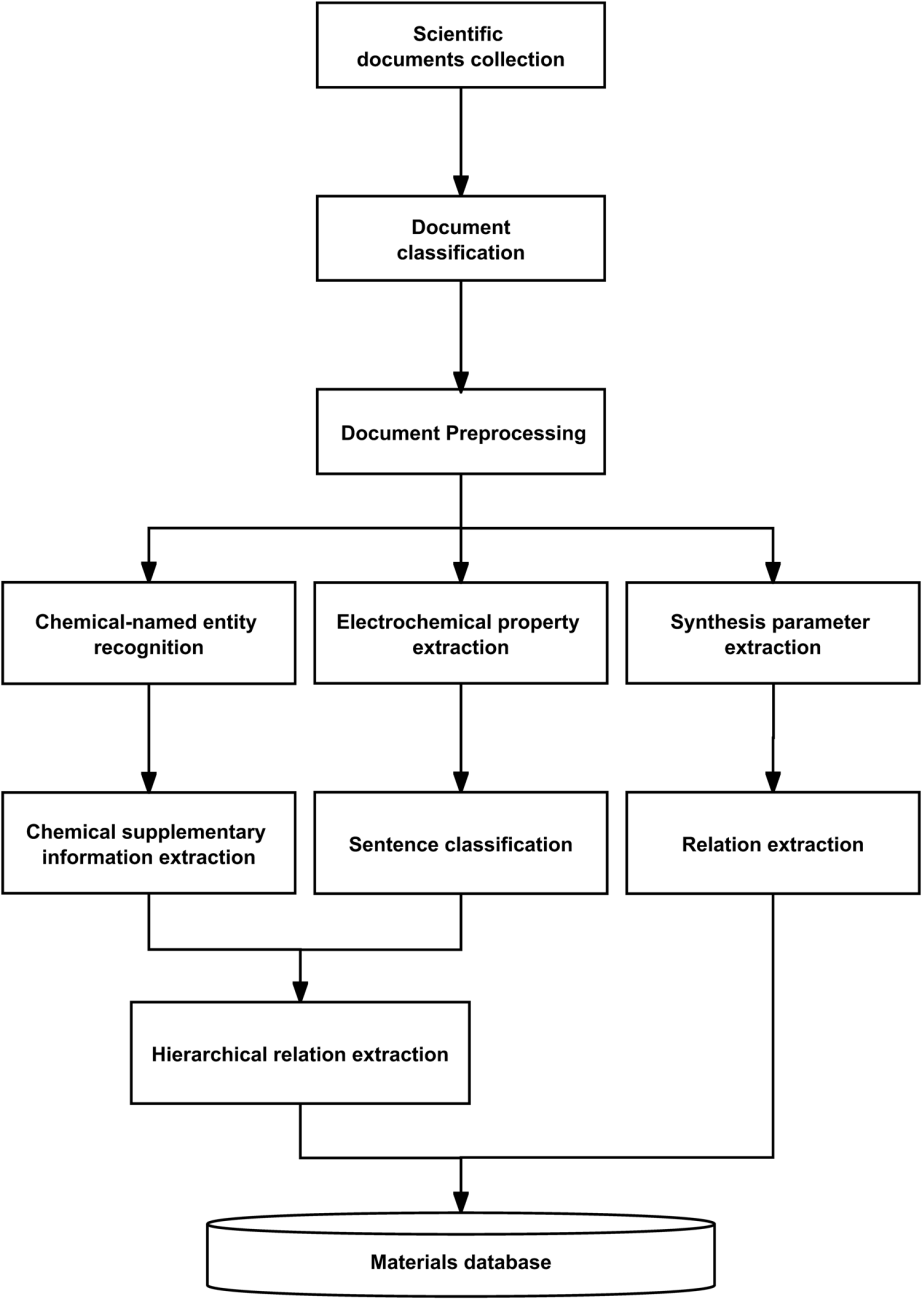
Schematic of the workflow of the automated text mining pipeline.

As Fig. 1 shows, our automated text mining pipeline for transition metal layered cathode materials involves several stages as follows:

Firstly, we use document classifiers to filter out scientific articles in extensible markup language (XML) and hypertext markup language (HTML) formats that contain the topics we are interested in. The purpose of document classification is to accurately grasp the topic of the article (see in **Document classification**).

Secondly, document preprocessing was performed on the original archive corpus in order to generate a complete and standardized document record and filter out irrelevant information, including paragraph classification and text preprocessing. Paragraph classification aims to isolate paragraphs involving different topics for different following tasks. Text preprocessing aims to unify the description of chemical expressions and related properties (see in **Document preprocessing**).

Thirdly, NER is performed, which includes chemical named entity recognition (CNER), chemical supplementary information extraction (CSIE), electrochemical property extraction, and synthetic parameter extraction. The NER method aims to extract the named entities, properties, and property values of cathode materials from English texts. CSIE refers to the process of enhancing the information associated with identified chemical entities and unifying the sequence of these entities. This process primarily involves identifying abbreviation definitions and determining variable values (see in **Named entity recognition**).

Fourthly, relation extraction is carried out. Additionally, in the context of electrochemical property relation extraction, sentence classification is utilized to identify target sentences associated with different electrochemical properties. Relation extraction gives specific tuple relations of element contents and properties and interdependency parsing is used to find links between specific materials and their property data fragments.

Finally, the extracted tuple entities containing the digital object identifier (DOI) of the article, the year of publication, the layered cathode material entity and its abbreviation, property and property values are automatically compiled into a highly structured format to form the layered cathode materials for SIBs database.

### Corpus of papers

**Fig. 2:**
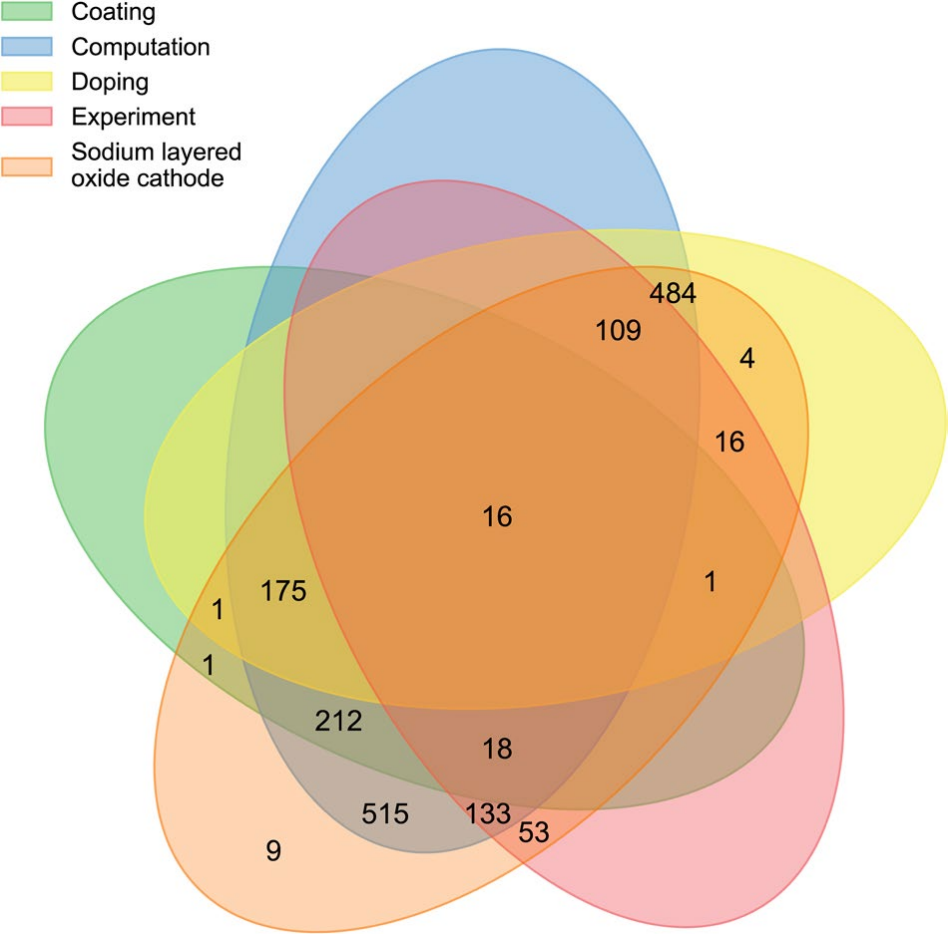
Venn diagram of corpus label distribution.

**Fig. 3:**
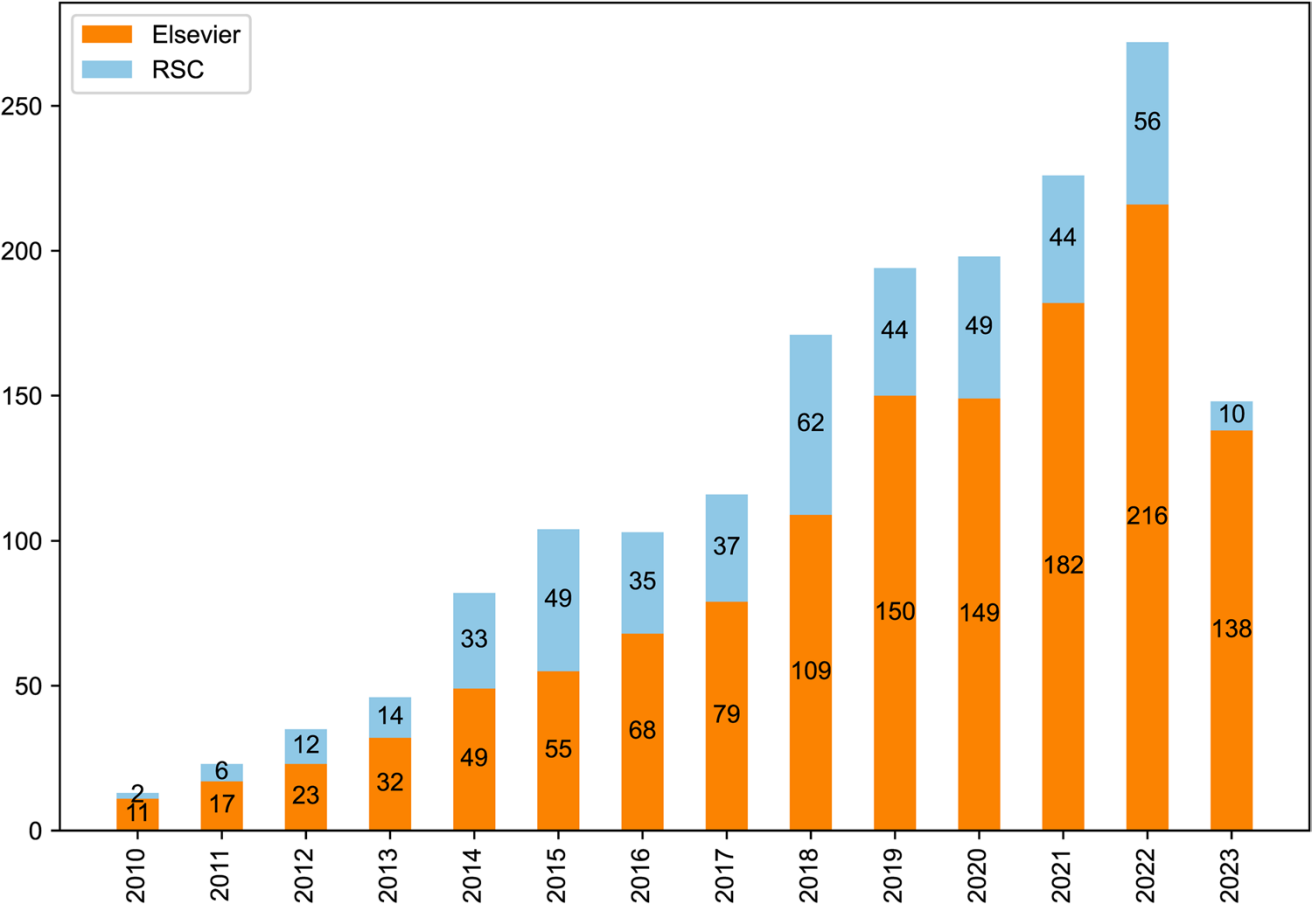
Stacked bar chart: the number of published articles about Sodium layered oxide cathode over the last 14 years. The vertical axis represents the number of journal articles related to the topic of Sodium layered oxide cathode.

We found that 29 manually annotated articles containing the label “Sodium layered oxide cathode” in the original dataset were not successfully identified, and they were added to the final corpus, resulting in a total of 1747 articles. Fig. 2 shows the distribution of research topics of articles in the sodium layered cathode material corpus, of which the maximum number of articles in pure material experimental synthesis research was 515 (about 29.48%). There were 484 articles (about 27.70%) and 212 articles (about 12.14%) studying materials by doping and coating modification experimental methods, respectively. It is worth noting that the number of research articles combined with computational science is about 346 (about 19.81%), which indicates that computational science plays an important role in the comprehensive research of sodium layered cathode materials. The corpus of this study contains 1702 articles on the research of sodium layered cathode materials from 2010 to June 2023 from Elsevier and RSC (the reason for the difference from the above 1747 is that the datasets contain articles before 2010). Fig. 3 shows the development trend of the number of articles on sodium layered cathode materials published by different publishers in the past 14 years. In general, with the rise of SIBs, the research on layered cathode materials of SIBs has been paid more and more attention by scholars.

### Named entity recognition

**Fig. 4:**
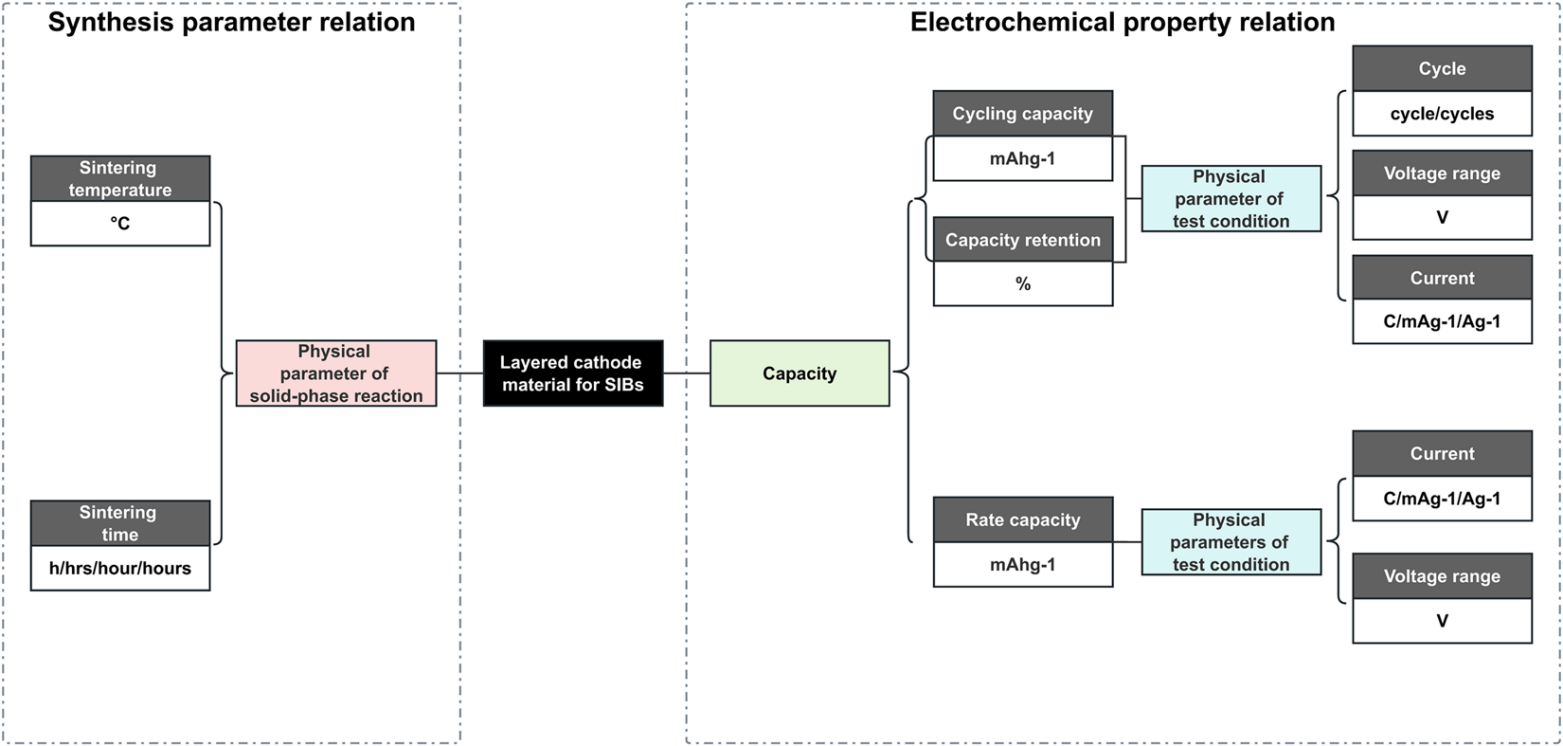
Cathode material property relationship network.

In the literatures of layered cathode materials, the relationships between chemical entities and their properties to be extracted can be presented as a complex ternary tuple <chemical name, properties, physical parameters>. As shown in Fig. 4, chemical names encompass various forms of cathode materials for SIBs such as chemical formulae (e.g., Na_0.9_Mn_0.52_Fe_0.28_Cu_0.2_O_2_ and NaNi_0.5-x_Mn_0.3_Ti_0.2_Sb_x_O_2_), abbreviations (e.g., CFM-Cu and NMTSb_x_), or pronoun phrases (e.g., this material, or even this sample when x = 0.15). The properties comprise the name, value, and unit of the material’s property. In addition, physical parameters encompass both synthesis parameters and test condition parameters for measuring electrochemical properties. The synthesis parameters include material sintering temperature and sintering time, which are crucial factors in the fabrication process of the material. These parameters directly affect the physical properties and performance of the synthesized material. On the other hand, the test condition parameters, such as current density and voltage range, are important when evaluating the performance and behavior of the material in a specific application or testing setup. These parameters provide insights into how the material responds to different levels of electrical current and voltage, which are essential for understanding its electrochemical performance. Considering both synthesis parameters and test condition parameters is crucial for a comprehensive analysis of the material’s physical characteristics and its suitability for specific applications. In this work, a total of 1747 documents of layered cathode materials for SIBs were obtained through automatic document classification. After document preprocessing, a NER system based on the CNER module of ChemDataExtractor version 2.1 and heuristic rules was explored. Here, we illustrate the NER system, which provides a sequence of entities for subsequent relation extraction, as follows.

#### Chemical named entity recognition and chemical supplementary information extraction

CNER is a fundamental task in IE of material literature^19,20^. However, accurate location of the entity boundaries by chemical entity recognition models is challenging due to the wide variety of materials, the flexible formation of chemical entities, and the limited availability of labeled data for model training. In the case of research papers focusing on layered cathode material systems, the presence of chemical formulas with stoichiometric and elemental variables, as well as chemical formula abbreviations (e.g., NaNi_0.5-x_Mn_0.3_Ti_0.2_Sb_x_O_2_, O3-NaNi_0.45_Mn_0.3_Ti_0.2_M_0.05_O_2_, and NNMO), often leads to increased uncertainty in subsequent relation extraction. Therefore, CSIE becomes particularly important to accurately identify the values represented by these variables and the definitions of the abbreviated formulas.

To address this challenge, a hybrid CNER post-processing is proposed in this study. This method combines the domain knowledge of cathode materials with the text2chem (https://github.com/CederGroupHub/text2chem) toolkit, a Regex-based text parser developed by Kononova *et al*., can convert chemical terms and entities into chemical data structures. It builds upon the CNER module of ChemDataExtractor to take advantage of the supplementary information provided in parentheses after the entities (e.g., “(x = 0.03,0.05,0.07)” and “(M = Nb/Mo/Cr)”). The proposed approach aims to provide a sequence of entities consisting of defined elements for subsequent relation extraction tasks.

#### Identify the values of the variables

The tokens labeled as chemical entities by ChemDataExtractor are parsed with text2cem to cover multiple possible specific materials corresponding to a chemical formula with variables. Specifically, when stoichiometric number variables such as x, y, z, and element variables such as M (metal), and TM (transition metal) are found in the chemical entity, multiple stoichiometries or elements are extracted from a single material mention. For instance, “O3-NaNi_0.45_Mn_0.3_Ti_0.2_M_0.05_O_2_ (M = Nb/Mo/Cr)” is converted to “O3-NaTi_0.2_Nb_0.05_Mn_0.3_Ni_0.45_O_2_”, “O3-NaTi_0.2_Mo_0.05_Mn_0.3_Ni_0.45_O_2_”, and “O3-NaTi_0.2_Cr_0.05_Mn_0.3_Ni_0.45_O_2_”. Similarly, “NaNi_0.5-x_Mn_0.3_Ti_0.2_Sb_x_O_2_ (x = 0.03, 0.05, 0.07)” is converted to “NaTi_0.2_Mn_0.3_Ni_0.47_Sb_0.03_O_2_”, “NaTi_0.2_Mn_0.3_Ni_0.45_Sb_0.05_O_2_”, “NaTi_0.2_Mn_0.3_Ni_0.43_Sb_0.07_O_2_”.

#### Abbreviation definitions

Chemical formulae abbreviations are related to full chemical formulae based on their adjacency to parentheses and naming verbs. This approach is inspired by the algorithm for identifying abbreviation definitions in biomedical text by Schwartz and Hearst^21^. Usually, in an article, a chemical formula and its abbreviation appear in two types of scenarios: (a) *long-form* ‘(‘ *short-form* ‘)’ and (b) *long-form* … *naming-verb* … *short-form*. The main difference is the possible location of the *short-form*, with pattern (a) in parentheses and pattern (b) after the naming verb. Here, the *long-form* refers to chemical entities, the *short-form* indicates chemical formula abbreviation, and the *naming-verb* includes “marked”, “denoted”, “named”, etc. In practice, *short-form* and *long-form* are often interchangeable.

In detail, the process of extracting abbreviations and their definitions from cathode material text involves two main subtasks. The first subtask is the extraction of < *long-form*, *short-form* > pair candidates from the sentence. Both the *short-form* and *long-form* are derived from tokens labeled as chemical entities by ChemDataExtractor. Tokens successfully parsed by text2chem are marked as *long-form*, while tokens with two consecutive uppercase letters are marked as *short-form*. Once these steps are completed, a list of *short-form* candidate words for the *long-form* is generated. The subtask is to choose the appropriate subset of words. The main idea is to count the number of uppercase characters in both the long-form and short-form. If the number of repetitions is not less than two and the uppercase letters in the short-form appear in the long-form, a successful match is determined. For example, given the input string “O3-NaNi_0.45_Mn_0.3_Ti_0.2_M_0.05_O_2_ (M = Nb/Mo/Cr, abbreviated as NMTNb, NMTMo and NMTCr, respectively)” the following mapping relationships are returned: (‘NMTNb’, ‘O3-NaTi_0.2_Nb_0.05_Mn_0.3_Ni_0.45_O_2.0_’), (‘NMTMo’, ‘O3-NaTi_0.2_Mo_0.05_Mn_0.3_Ni_0.45_O_2.0_’), (‘NMTCr’, ‘O3-NaTi_0.2_Cr_0.05_Mn_0.3_Ni_0.45_O_2.0_’) in a list.

This algorithm is applied to all sentences in paragraphs to produce a list of mappings between chemical formula abbreviations and their corresponding chemical formula. These mappings are then used to transform chemical formula abbreviations in the literature extraction records into more informative chemical formulas corresponding to elemental compositions.

#### Synthesis parameter and electrochemical property extraction

There are two categories of information that are the most important to describe layered cathode materials for SIBs: electrochemical performance and synthesis method. The former is usually evaluated using cycling performance and rate performance. The latter can be covered using sintering temperature and sintering time because layered cathode materials are typically synthesized using a simple high-temperature solid-phase method^22^.

The paragraphs to extract synthesis parameters and electrochemical properties are derived from the previous paragraph classification process. To cover the properties mentioned in the sentences as comprehensively as possible, we have customized multiple matching rules in the form of regular expressions, as Supplementary Table [1] summarizes. In particular, three common units of current density properties, namely C, mA g-1, and A g-1, are considered in this study. To ensure consistency, conversion expressions between different units of current density that may appear in the article are taken into account. For example, conversions such as “1 C = 150 mAg-1”, “20 mAg-1 (0.1 C)”, and “0.2 C (50 mAg-1)” are considered to standardize the units of current density to mAg-1. To precisely determine capacity retention, we introduce the property descriptors “retention” and “capacity”. For the number of cycles, descriptors such as “first” and “initial” are considered to indicate the initial number of cycles. Subsequently, the identified character sequence covered by the aforementioned pattern is tokenized to extract the property values.

The final property extraction is recorded as a key-value pair, where the key consists of the property name with its unit, and the value represents the corresponding property value. To illustrate the property extraction process, consider the following example sentence: “After 300 cycles, a discharge capacity of 47 mAh g-1 is obtained, only equal to 66% of the initial capacity.”^23^. Based on the provided sentence, the extracted property values can be recorded as a key-value pair in the following format “{‘cycle’: [300, 1], ‘capacity: mAhg-1’: [47], ‘retention: %’: [66]}”.

### Relation extraction

Extracting multiple relationships from cathode material papers is indeed a challenging task. It involves the extraction of two types of property relationships, as shown in Fig. 4. The first type encompasses electrochemical property relationships, which can be further classified into cycling performance property relation and rate performance property relation, they are tabulated as complex tuples, <cathode material, the number of cycles, capacity/retention, current density, and voltage range> and <cathode material, capacity, current density, and voltage range>, respectively. The second type pertains to synthetic parameter relationships, which can be represented as a binary tuple consisting of sintering temperature and sintering time. Multiple cathode materials, their specified properties, and corresponding property values are often reported in separate sentences. Alternatively, multiple properties and their corresponding values may be reported for a single cathode material. These complexities pose obstacles for relation extraction, especially when working with a limited corpus of cathode materials for SIBs. Hierarchical relation extraction supervised algorithms require more than ~70,000 labelled sentence samples^24^. Even semi-supervised methods require a certain number of labelled samples as seeds to start learning. It is worth noting that most relation extraction systems primarily focus on extracting relations at the sentence level and do not consider relations that span across multiple sentences or paragraphs^25^. In this study, we present a novel order and distance-based algorithm that that does not rely on labelled samples for relationship extraction. Our proposed approach considers relationships across sentence levels. In feature-based relationship extraction methods, the number of words and word sequences between entities and properties can be used as the main syntactic features^26^. Thus, the number and order of entity occurrences, along with the distance between entities provide a basis for assessing relation dependencies.

Specifically, the algorithm we propose starts from the target sentence, where the target sentence for different tasks of relation extraction is determined by the combination of entities appearing in the sentence. Sentences mentioning number of cycles and capacity/retention are used for cycling performance property relation extraction, while those mentioning capacity and current density but not number of cycles are used for rate performance property relation extraction. Once the target sentence is identified, the relationship tuples for extraction are determined. Subsequently, matching commences from entities with stronger dependency relationships, considering the quantities of the entity sequences, denoted as n1 and n2.

1. **Shortest Character Distance:** This addresses cases where specific entities are absent in the sentence (n1 = 0 or n2 = 0). It focuses on instances where entities like cathode material or voltage range is missing, and fill it by finding the closest entity to the sentence head index based on character distance in the preceding text.
2. **Sequential Matching:** It tackles situations where n1 = n2 in the sentence, and the matching order is determined by the order of the two entity sequences. Particularly, when either n1 or n2 equals 1, the entity with a quantity of 1 is multiplied to match n1 = n2 based on a greedy rule. Here, we haven’t emphasized the remaining cases where n1 is not equal to n2, as they occur less frequently due to the precise nature of battery literature.

Once the matching of the two entities is completed, several tuples with associated relationships are obtained. These tuples are treated as new entity sequences and are matched again with other entities in the target tuple, repeating the process until the target tuple is filled as comprehensively as possible.

For matching cycling performance property relation, the process begins with the number of cycles and capacity/retention, and then sequentially matches with cathode materials, voltage range, and current density.

For matching rate performance property relation, matching starts with current density and capacity, and then sequentially matches with cathode materials and voltage range.

**Fig. 5:**
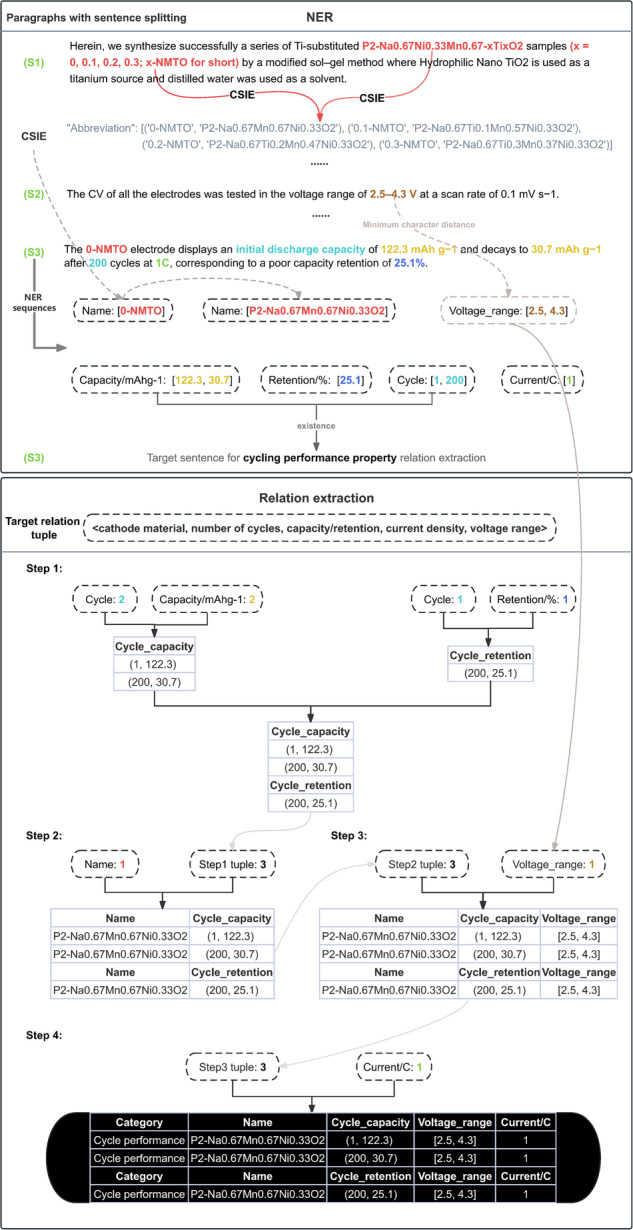
Multivariate hierarchical relation extraction for the electrochemical property.

Fig. 5 illustrates a complete information extraction process, where sentences S1, S2, and S3 are dispersed throughout the paper^27^ with certain contextual relationships, and S3 is the target sentence.

In the NER stage, CSIE establishes linkages for contextual cathode material entities and their abbreviations. The identified entity sequence in S3, including the number of cycles and capacity/retention, provides the descriptive intent for the sentence, used for subsequent extraction of cycling performance property relation.

In the relation extraction stage depicted in the Fig. 5, numerical values are used to describe the lengths of entity sequences. In Step 1, matching is performed for the relationships involving the number of cycles and capacity/retention. It’s worth noting that due to the comparative nature of capacity retention, it cannot be matched with the initial cycle, hence in this step, both lengths of entity sequences are the same, and sequential matching is executed. In Step 2, based on the tuples matched in the first step, further matching with cathode material entity is conducted, establishing linkages with preceding supplementary information, and sequential matching is performed based on quantity relationships. In Step 3, matching with voltage ranges is performed based on the results of the second step. Since the voltage range entity sequence is missing in S3, the shortest character distance links to the voltage range extracted from S2, followed by sequential matching based on the greedy approach. Step 4 follows a similar logic, resulting in the complete target tuple.

**Fig. 6:**
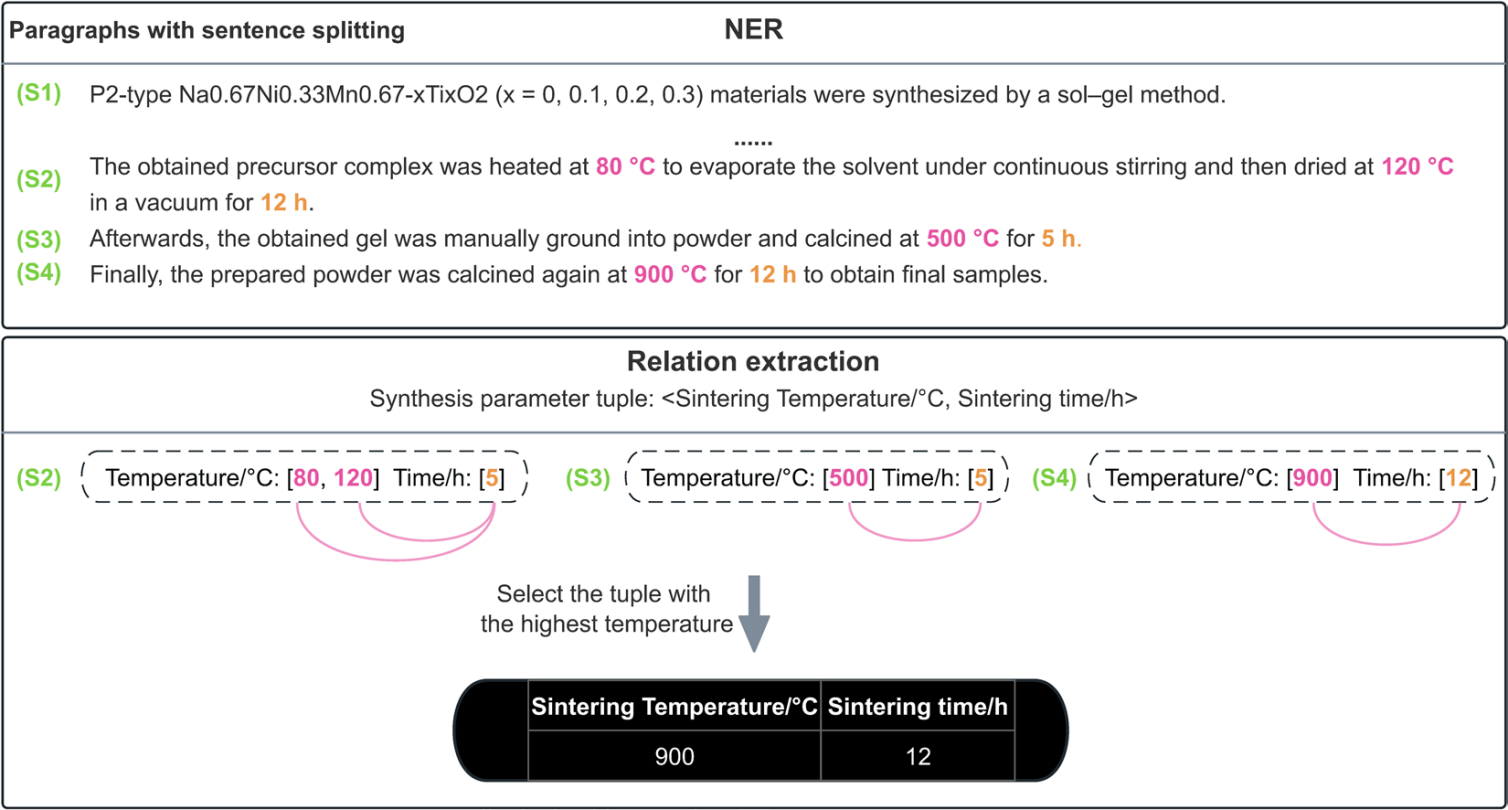
Synthesis parameter binary relation extraction.

In the synthesis parameter binary property relation, which can be considered as the most basic scenario for this algorithm to handle, a greedy-based sequential matching strategy is used to match two sequences of entities, as depicted in Fig. 6. This strategy aims to generate pairs of sintering temperature, sintering time> that are present in the experimental paragraphs of the article. Ultimately, the highest sintering temperature in the binary relation is selected as the synthesis parameter of the material investigated in the article.

### Dataset overview

Using our pipeline, we successfully extracted 5265 property records of layered cathode materials for SIBs from a total of 1747 articles. Among these records, 3557 correspond to cycling performance properties, while 1708 pertain to rate performance properties.

**Fig. 7:**
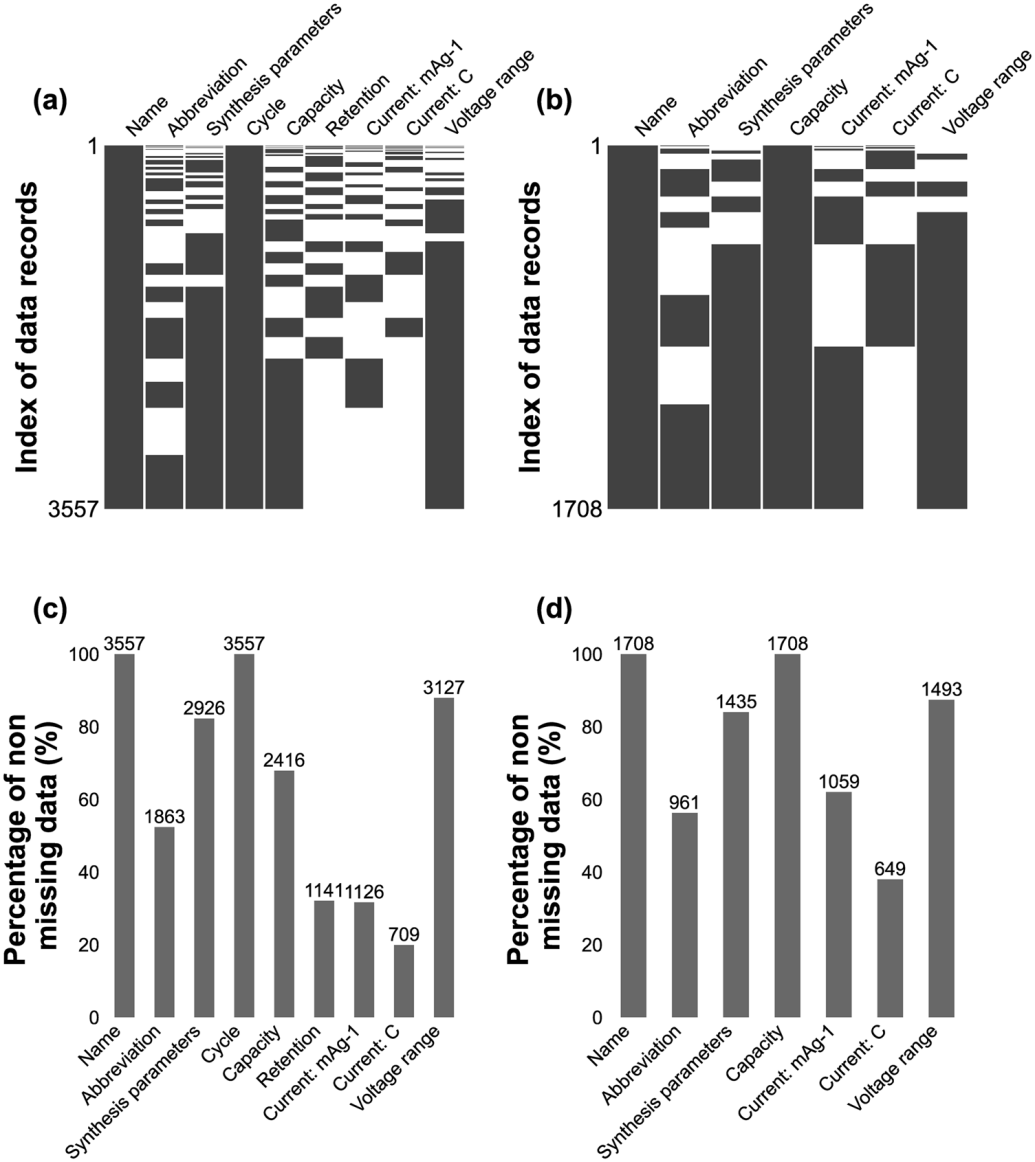
Visualization of missing values in the cycling performance and rate performance. (**a**) Distribution of missing values in the cycling performance data. (**b**) Distribution of missing values in the rate performance data. (**c**) Percentage of non-missing values in cyclic performance data. (**d**) Percentage of non-missing values in cycling performance data.

Fig. 7 visually represents the distribution and percentage of missing values in the records, where each column corresponds to a specific property. Fig. 7a,b illustrate the distribution of missing values of in the cycling and rate performance data among records of materials, respectively. In this representation, each row in the figure represents a single piece of data and the presence of a blank cell indicates missing values of the property corresponding to the column. Notably, the sintering temperature and sintering time are merged into the column of “Synthesis parameters”, so are the upper and lower test voltage are into “Voltage range” in order to show the data distribution more clearly. The current density property is presented in units of mAg-1 or C, depending on the specific record. Regarding the property of cycling performance, each row of data contains the retention or capacity value of the material for a specific number of cycles. On the other hand, for records related to rate performance properties, each row of data represents the material’s capacity at a particular current density. Fig. 7c,d provide an overview of the proportion and count of missing properties in the cycling and rate performance records, respectively. In the cycling performance records, there is a notably high number of missing values for the current density attribute. This can be attributed to the dispersed nature of the descriptions of test condition parameters of cycling performance in literatures, particularly when current density is described. Indeed, this observation underscores the challenge posed by the diverse patterns of descriptions of current density test in battery literature in natural language, which complicate the task of accurately extracting relevant information. Specifically, challenge may arise from differences in units, formatting, or the use of various terminology across different sources. Consequently, it becomes crucial for future work to develop robust extraction methods that can effectively handle these diverse patterns and ensure accurate and reliable information extraction from battery literature.

**Fig. 8:**
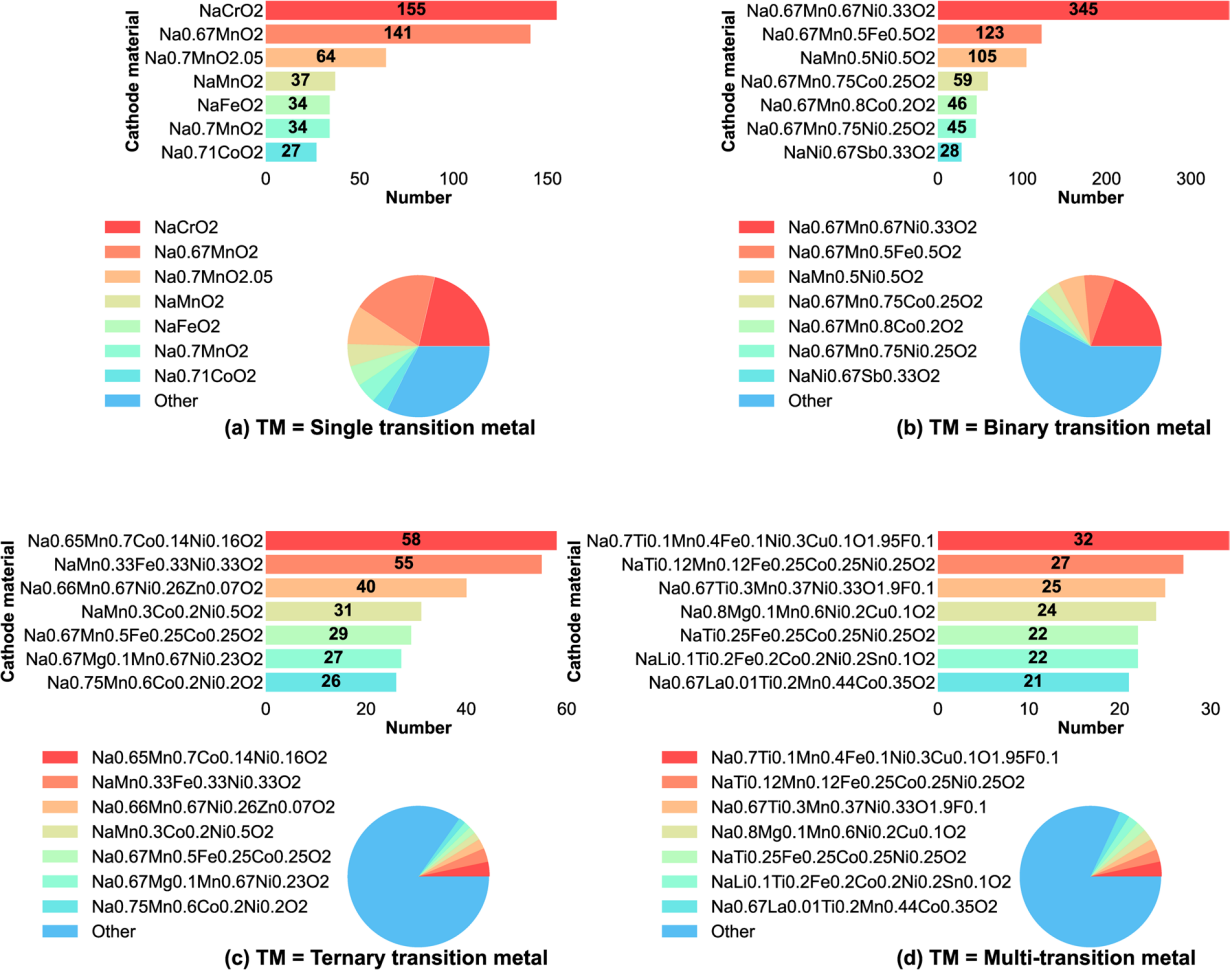
Distribution of layered oxides Na_x_TMO_2_ (TM = single, binary, ternary or multi-transition metal) cathode materials and its top seven representatives.

Among the 5265 unique data records, a total of 592 unique layered oxide cathode materials were identified. Fig. 8 provides an overview of the chemical distributions of layered oxides with the general formula NaxTMO_2_, where TM represents transition metals. These transition metals can be single elements or mixtures of two, three, or multiple elements. Additionally, Fig. 8 specifies the distribution of the top seven representative materials for each type of layered oxide material. This facilitates the identification of the most popular layered cathode materials in recent studies. For example, the figure highlights the most popular single transition metal layered cathode materials, such as NaxCrO_2_, NaxMnO_2_, and NaxFeO_2_. It also showcases the distribution of binary transition metal layered cathode materials like Na_x_Mn_1-y_Ni_y_O_2_, where M represents elements such as Ni, Fe, or Co. Furthermore, the figure illustrates the distribution of ternary transition metal layered cathode materials, including Na_x_Mn_1-y-z_M_y_Ni_z_O_2_, where M can be Co, Fe, or Zn. Lastly, it showcases the distribution of multi-transition metal Mn-based layered cathode materials that have been studied extensively in recent years.

### Model Performance

#### Multi-label text classification

Table 1 presents the combined Precision, Recall, F1 score, as well as Micro average and Macro average results of the model’s binary document classification for each label on the test set. The binary classification results indicate that the model achieves the highest overall performance on the “Experiment” label, with an F1 score of 95.86%. However, the model shows a relatively poorer performance on the “Coating” label, with an F1 score of 74.33%. This discrepancy can be attributed to differences in the model’s learning effect due to the uneven distribution of training samples.

**Table 1:** Evaluation of multi-label text classification model.

Predicted Label		Precision	Recall	F1 score
*Coating*		68.85%	80.77%	74.33%
Computation		84.78%	88.64%	86.67%
*Doping*		89.99%	96.92%	93.33%
*Experiment*		97.04%	94.71%	95.86%
*Sodium layered* oxide *cathode*		83.78%	85.32%	84.54%
**Aggregate Metric**	*Micro average*	**88.39%**	**90.79%**	**89.58%**
	*Macro average*	84.89%	89.27%	86.95%

Specifically, the “Coating” label has a limited number of training samples, resulting in a weaker performance from the model on this label. In contrast, the ‘Experiment’ label benefits from a larger number of training samples, allowing the model to learn more effectively and perform better in its prediction (F1 score of 95.86%). This distribution of labeled data also confirms that experimental synthesis is the predominant research approach in the field of traditional materials.

Furthermore, the Aggregate Metric evaluation results demonstrate that the F1 scores for all models reach 85%. Additionally, the models outperform the Macro average in all indicators of the Micro average. This can be observed from Eqs. (8), (9), and (10), which indicate that the Macro average, being a simple arithmetic average, does not consider the issue of sample distribution. As a result, the scores may be biased towards labels with significant differences in values.

In conclusion, the model can be successfully utilized for the screening of articles on layered cathode materials for SIBs and for analyzing the research methodologies employed in these articles.

#### Paragraph classification

In this study, a total of 50 documents were randomly selected from the document collection. The categorized paragraphs within these documents were manually checked. The performance of the categorization process was evaluated using precision, recall, and F1 score metrics, as demonstrated in Table 2.

**Table 2:** Precision, Recall and F1 Score of the paragraph classification.

Paragraph category	Precision	Recall	F1 score
*Introduction*	100%	100%	100%
*Experiment*	100%	91.99%	95.83%

#### Abbreviation definitions

The evaluation of abbreviation detections in this study was primarily based on the matching of binary relation pairs between sodium layered cathode materials and their corresponding abbreviations. Correct matches were designated as TP, while incorrect matches were designated as FP. Additionally, binary relation pairs that were not recalled were considered FN. Using these criteria, abbreviation detections was performed on 705 sentences randomly selected from 98 articles (totaling approximately 16,442 sentences). Furthermore, to assess the performance of the improved module within a specific domain, the abbreviation detection module in “ChemDataExtractor” was utilized as a control. The precision, recall, and F1 scores obtained from the final experiments are provided in Table 3, with precision at 98.02%, recall at 87.94%, and F1 score at 92.71%.

**Table 3:** Precision, recall, and F1 score of the sodium layered cathode material entity abbreviation definitions.

Methods	Precision	Recall	F1 score
*This work*	98.02%	87.94%	92.71%
*ChemDataExtractor 2.1.2*	95.45%	45.32%	61.46%

#### Relation extraction

To ensure the reliability of our pipeline extraction, we conducted a verification process using a test set comprising a total of 10 papers^28–37^ from the databases of this study (RSC, Elsevier) and 12 papers^38–49^ from other journals (Nature Communications, Advanced Materials, American Chemical Society, Small) were randomly selected. These papers were available in XML/HTML format.

For the verification process, researchers manually reviewed the original article text and labeled the key information related to the tasks specified in Table 4. The pipeline output was compared with the manually compiled standard output, and Precision, Recall, and F1 scores were calculated for various tasks based on this comparison.

**Table 4:** P (Precision), R (Recall), and F1 (F1 scores) on different datasets for the six main tasks: Abbreviation definitions, Synthesis parameter relation extraction, cycling performance sentence classification, Rate performance sentence classification, Cycling performance relation extraction and Rate performance relation extraction.

Task			Abbreviation definitions	Synthesis parameter relation extraction	Cycling performance sentence classification	Rate performance sentence classification	Cycling performance relation extraction	Rate performance relation extraction
**Test datasets**	*12 articles from other journals*	**P**	94.74%	100%	90.91%	92.68%	69.84%	90.24%
		**R**	78.26%	88.89%	96.15%	84.44%	68.75%	74%
		**F1**	85.71%	94.12%	93.46%	88.37%	69.29%	81.32%
	*10 articles from RSC and Elsevier*	**P**	93.75%	100%	96%	93.55%	85.96%	96.88%
		**R**	93.75%	100%	100%	100%	89.91%	74.70%
		**F1**	93.75%	100%	97.96%	96.67%	87.89%	84.35%
	*Total (22 articles)*	**P**	94.29%	100%	93.85%	90.54%	80.23%	93.15%
		**R**	84.62%	94.12%	98.39%	90.54%	82.08%	74.32%
		**F1**	89.19%	96.97%	96.06%	90.54%	81.14%	82.67%

**Table 5:** Abstract categories and their definitions.

Category	Definition
Coating	This article presents the properties of the materials studied by coating modification.
Computation	The research on the intersection of materials science and computer science mainly includes computational simulation, computer design of materials and machine learning.
Doping	In this article, the properties of the material are studied by doping.
Experiment	The article has material synthesis experiments.
Sodium layered oxide cathode	The research area of this article is about layered cathode oxide materials.

Table 4 provides an overview of the Precision, Recall, and F1 scores for the four primary tasks in relation extraction: Abbreviation definitions, Synthesis parameter relation of material synthesis, Cycling performance sentence classification, and Rate performance sentence classification. Additionally, the overall Precision, Recall, and F1 scores of the two comprehensive tasks, Cycling performance relation extraction and Rate performance relation extraction, are also displayed. In these evaluation metrics, an entire data record extracted is treated as a single unit. If a record is totally or partially missing compared to the manually extracted information, it is considered as a false negative. If the record is partially incorrect, it is considered as a false positive. If and only if the record is entirely correct, it is considered as a true positive.

**Fig. 9:**
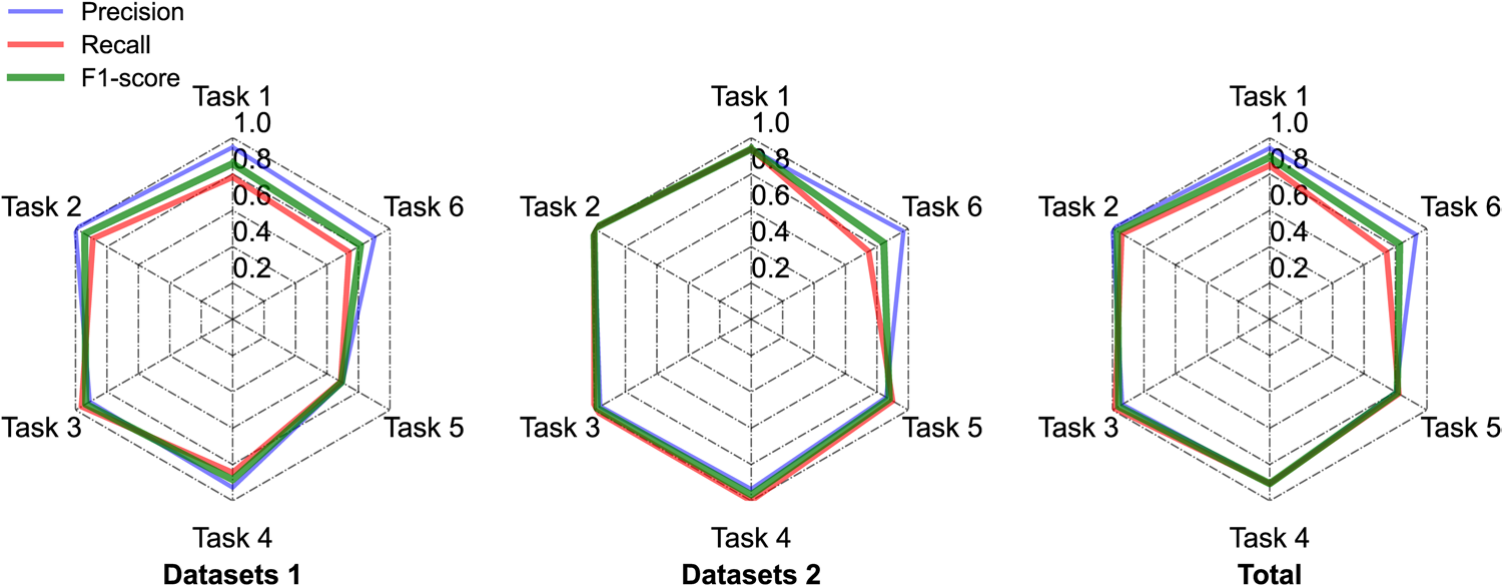
Radar plot: Performance of different tasks on different datasets.

Fig. 9 illustrate the performance of each relational extraction task across different datasets. The Precision scores consistently remain high for the four base tasks, and in some cases, there are even no false positives in the test set. This success can be attributed to the hybrid NLP approach employed by the pipeline, which leverages domain knowledge specific to cathode material for SIBs. This approach is well-suited to the structured language and format commonly found in scientific articles on sodium layered cathode materials. The accurate matching of Abbreviation definitions is particularly noteworthy, as it serves as a crucial prerequisite for successful extraction of cycling performance and rate performance properties of sodium layered cathode materials. However, the scores for the Cycling performance relation extraction and Rate performance relation extraction tasks are lower compared to the four basic tasks. This can be attributed to the inherent complexity of these tasks.

**Fig. 10:**
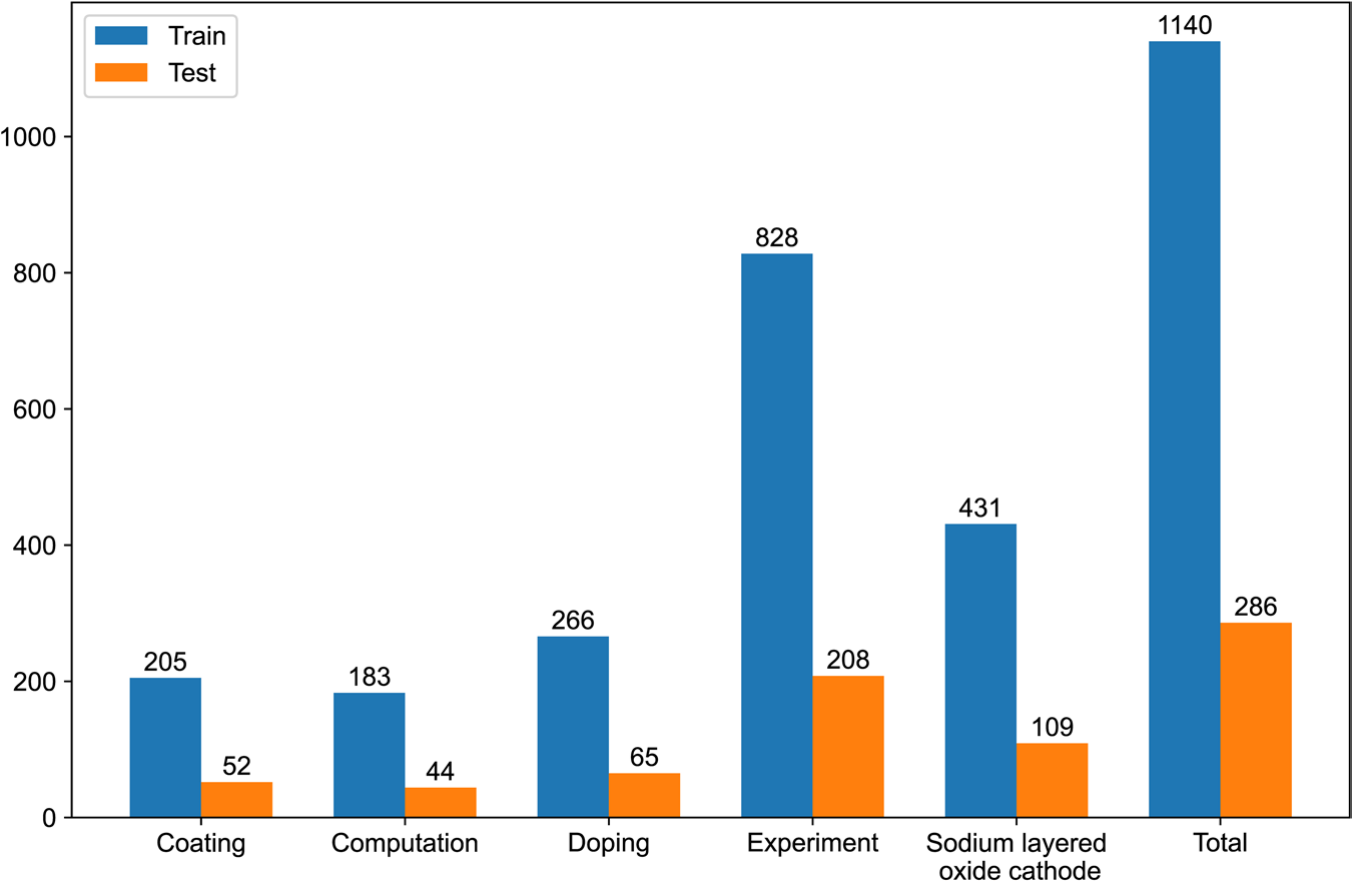
Multi-label classification datasets.

Specifically, the performance of Cycling performance relation extraction is notably poor in Test Dataset 1, primarily due to challenges in Abbreviation definitions and Synthesis parameter relation. The low recall in Abbreviation definitions can be attributed to irregularities in material abbreviation naming. For instance, in the text^42^, “Na0.8” is used as the abbreviation for “P2-Na_0.8_Ni_0.1_Mn_0.8_Fe_0.1_O_2_” which leads to incomplete identification of chemical entities by the pipeline. Additionally, the low recall in Synthesis parameter relation of material synthesis can be attributed to a few articles^39^ that primarily focused on investigating the effect of phase structure on electrochemical properties at various synthesis temperatures. In contrast, the study at hand specifically focuses on the time corresponding to the highest synthesis temperature. These factors contribute to a degradation in performance. In summary, there was a slight decrease in accuracy and recall when moving from internal journals to external combinations of journals from different publishers. This can be attributed to the greater variety of formats and non-standard representations encountered in the different journals. However, despite this challenge, the overall F1 score performance of the pipeline on both data combinations remains above 81%, indicating robustness.

## Discussion

Our proposed method successfully collected a comprehensive database consisting of 5265 data records from 1747 articles, encompassing synthesis parameters, cycling and rate performance properties, along with relevant test condition parameters. The dataset we have constructed provides valuable insights into the characteristics of sodium layered cathode materials, aiding future research in this field. Furthermore, the key novelty of our methods is the enhancement of relation extraction across sentences, which presents a significant challenge within the realm of information extraction. It is realized by incorporating cross-sentence and even paragraph-level extraction to extract relations which cannot be captured by previous. As far as our knowledge extends, our information extraction pipeline represents a pioneering effort in the materials domain methods.

In detail, in the relation extraction stage, we proposed an effective heuristic multi-level relationship extraction algorithm. This algorithm incorporated information from the full text, particularly the chemical complementary information, including the detection of chemical formula abbreviation full names and variable values associated with chemical formulae, significantly enhanced entity information. Additionally, the extraction of performance properties at the sentence level was considered. As a result of these efforts, the relation extraction algorithm achieved average F1 scores of 81.14% and 82.67% for Cycling performance and Rate performance relation extraction in different journals, respectively, indicating strong robustness.

It is noteworthy that despite the primary focus of this study being on papers in XML/HTML format, our information extraction pipeline also has the potential to process papers in PDF format by using toolkits such as PDFMiner (https://github.com/euske/pdfminer) to convert them into plain text, thus ensuring the smooth extraction of information. Furthermore, our extraction strategy is not limited to the synthesis parameters, cycling performance, and rate performance properties of sodium layered cathode materials for SIBs. By adjusting the prompted elements in the CNER module to “Li”, our approach can be applied to the research of layered cathode materials for LIBs. Additionally, by incorporating a tailored CNER model for a specific battery cathode material field, it becomes possible to extract cathode material properties studied within that particular domain. This flexibility allows our extraction pipeline to be adapted and extended to various research areas, enhancing its applicability and potential for extracting valuable information from different types of texts.

However, there are problems that we have not yet solved and require further advancements, Firstly, manual intervention is necessary for data cleaning and refinement because of inevitable errors and duplicates among the automatically extracted records. Secondly, accurately capturing attribute values described as ranges, such as “from… to…,” and understanding their potential relationships with other attributes still remains a challenge. Thirdly, the extraction process did not account for relevant coating materials in the context of coating modification. These aspects are crucial for the development of cathode materials and should be incorporated to enrich the existing database.

## Methods

### Document classification

In literature searching only “sodium ion battery” was entered as the keyword to collect more articles that might be of interest. This search strategy led to many articles that included titles, abstracts, keywords, or articles in the main content that are similar to related topics. However, some of them focus on other materials such as nano-optoelectronic materials, metal catalytic materials, metal-organic skeletons (MOF), etc. Irrelevant articles in the corpus may decrease the accuracy of content mining. Therefore, we combine binary document classification and multi-label text classification (MLTC) on the abstracts to filter out irrelevant articles and obtain a set of articles of layered cathode material for SIBs as follows:

1. Binary document classification to classify whether a research paper is relevant to battery research or not. The battery document classification model BatteryOnlyBERT-uncased (accuracy up to 97.08%) fine-tuned by Huang *et al*.^50^ was used. 10565 articles from RSC and 30591 articles from Elsevier were retained.
2. Further, a MLTC task (Coating, Computation, Doping, Experiment, Sodium layered oxide cathode) was performed to refine the research method of the article according to the given abstract with a fine-tuned BatteryBERT-cased model. Definitions of classified abstract categories are shown in Table 5. 1140 manually annotated abstracts were used for training and tested on an additional 286 labeled abstracts (dataset label distribution is shown in Fig. 10). The articles with “Sodium layered oxide cathode” in the predicted label were screened out, and 458 articles from RSC and 1260 articles from Elsevier were retained based on 1).

### Document preprocessing

#### Paragraph classification

Among the dozens of paragraphs in an HTML/XML document, classification allows us to determine which paragraph contains the target property information to be extracted. In the following synthesis parameter and electrochemical property extraction, different sub-tasks would be performed on paragraphs of different categories or combinations of categories. A text extraction algorithm based on section headings was employed to classify the paragraphs into five categories: “Abstract”, “Introduction”, “Experiment”, “Metadata” and “Others”. Metadata sections such as “Acknowledgement”, “Reference” and “Conflict”, which do not contain inorganic materials, were excluded.

**Fig. 11:**
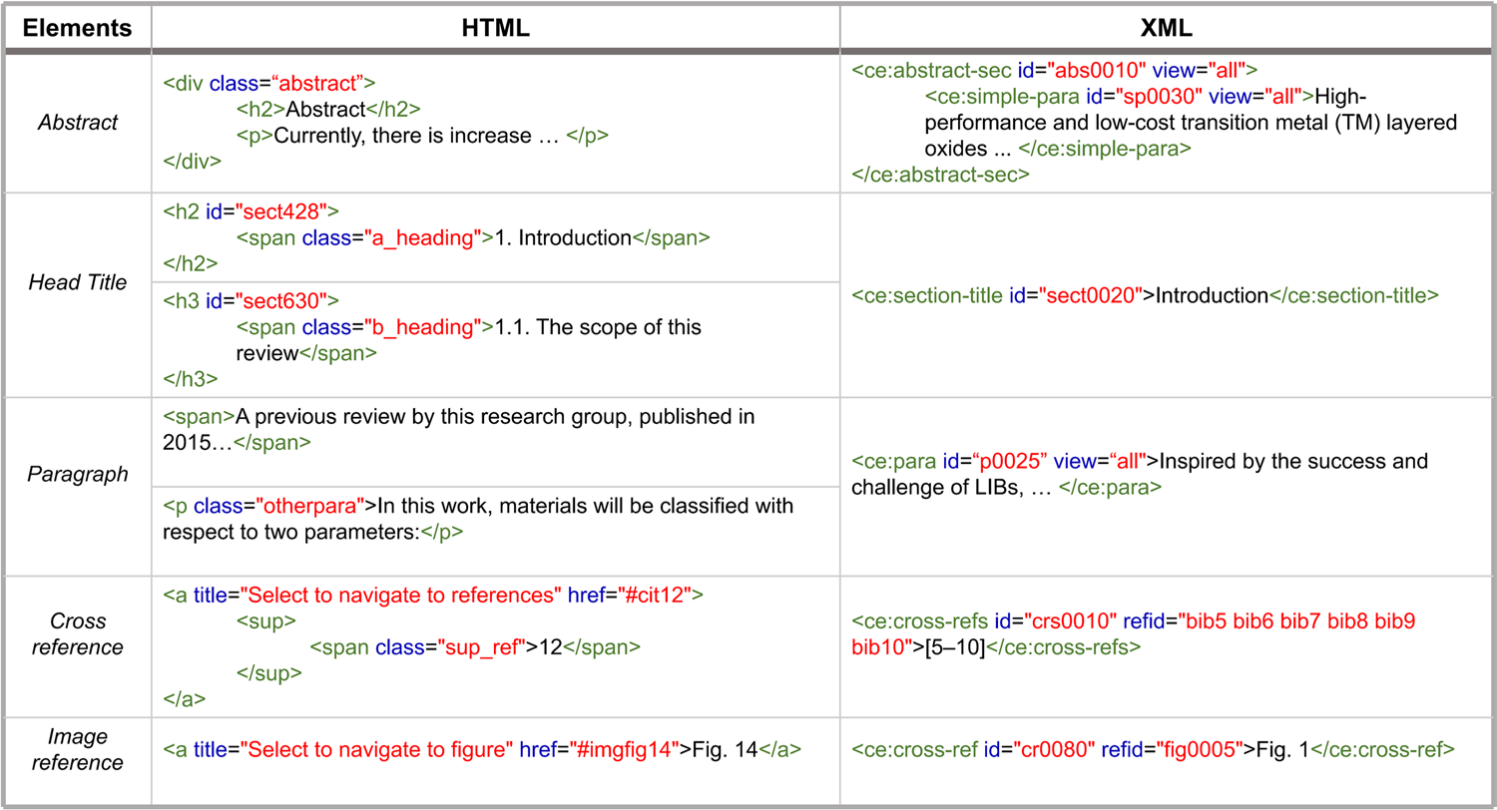
HTML/XML tag categories and their hierarchies.

Specifically, Python library “Beautifulsoup4” was utilized to locate various title tags (Fig. 11). Then the title texts were classified using customized regular expressions (Table 6). Afterwards, we parsed the plain text of all paragraph tags under the classified title tag to achieve paragraph classification. Within the paragraphs, boundaries are marked with “$$”, cross-references include image references and reference references, and the character “<CR>” is uniformly used instead of reference references, and “<FIG>” is used instead of image references.

**Table 6:** Classification and corresponding tasks of sections.

Section	Matching rules (Regular expression)	Tasks
*Abstract*	(?i:abstract)	document classification, CSIE, electrochemical property relation extraction
*Introduction*	(?i: intro)	CSIE
*Experiment*	(?i:prepar|Synthes|proce|experiment|Method|Material|Chemical|Fabrication|Composition)	CSIE, synthesis parameter relation extraction,
*Metadata*	(?i: Acknowledgement|Reference|Conflict|Author)	—
*Other*	—	CSIE, electrochemical property relation extraction

**Table 7:** Format of each data record: data class, data key label, description, data type.

Data class	Data key label	Description	Data type
*Metadata*	Doi	DOI of the article	String
	Year	Published date	Int
*Named Entity*	Name	Chemical formula of the material	String
	Abbreviation	Abbreviation of the material	String
	Elements	Elemental composition of the chemical formula	Object (dict)
*Synthesis parameter*	Temperature	Sintering temperature of the material	Float
	Time	Sintering time of the material	Float
*Performance category*	Category	Cycling performance or Rate performance	String
*Electrochemical property*	Cycle	Number of cycles	Int
	Capacity	Specific capacity of the cathode	Float
	Retention	Capacity retention rate of cathode	Float
	Current	Current density value	Float
	Voltage lower limit	Lower bound of the test voltage range	Float
	Voltage upper limit	Upper bound of the test voltage range	Float

To enhance the efficiency of our method, we have implemented a meticulous task allocation for various categories of paragraphs. Specifically, we have filtered out the paragraphs categorized as “metadata” while ensuring the participation of all paragraphs in CSIE. The paragraphs belonging to the “abstract” category are utilized for document classification and electrochemical property relation extraction, while those falling under the “experiment” category were used for synthesis parameter relation extraction. Furthermore, the remaining paragraphs, typically encompassing the results and discussion sections, were employed for electrochemical property relation extraction.

#### Text preprocessing

Chemical expressions, unicode characters and descriptions of related properties were normalized to eliminate inconsistency, facilitate the development of parsing rules for properties and improves the performance of CNER.

Firstly, the text was tokenized using ChemDataExtractor^51^. This involves splitting the raw text into sentences, followed by the splitting of each sentence into individual tokens. Then it was determined whether a token was a valid chemical formulae using pymatgen^52^ combined with regular expression and rule-based techniques. Finally, the identified valid chemical formulas were normalized to provide a unified chemical entity for subsequent literature mining of battery materials, involving the following steps:

1. Elements were ordered according to their electronegativity as defined in Table VI of the “Nomenclature of Inorganic Chemistry (IUPAC Recommendations 2005)”^53^ defined. For example, Na_0.67_Mn_0.7_Ni_0.15_Cu_0.15_O_2_ and Na_0.67_Mn_0.7_Cu_0.15_Ni_0.15_O_2_ were rewritten as Na_0.67_Mn_0.7_Ni_0.15_Cu_0.15_O_2_;
2. Redundant spaces and valence states were removed from chemical formulas. For example, Na[<SPACE> Ni_0.5_Co_0.2_Mn_0.3_]O_2_ (here <SPACE> indicates the space in chemical formulas) becomes Na[Ni_0.5_Co_0.2_Mn_0.3_]O_2_ and P2-Na_2/3_Mg(II)_1/4_Mn(IV)_7/12_Co(III)_1/6_O_2_ becomes P2-Na_2/3_Mg_1/4_Mn_7/12_Co_1/6_O_2_;
3. For chemical formulas containing Na and transition metal elements, redundant parentheses were removed. For example, Na[Ni_0.5_Co_0.2_Mn_0.3_]O_2_ becomes NaMn_0.3_Co_0.2_Ni_0.5_O_2_;
4. Elemental stoichiometric fractions were converted into decimals in chemical formulas. For example, P2-Na_2/3_Mg_1/4_Mn_7/12_Co_1/6_O_2_ becomes P2-Na_0.67_Mg_0.25_Mn_0.58_Co_0.17_O_2_;

In addition, Unicode characters that are used interchangeably are standardized, and all non-printing control characters were removed. For example, “–”, “⁃”, “-” were replaced as “-” and “\u0001”, “\u000e”, “\u001a” were removed. Furthermore, units of sintering temperature, sintering time, current density and capacity, which correspond to the most important property parameters of SIBs, were unified as “°C”, “h”, “mAg-1”, “Ag-1”, and “mAhg-1”, respectively, using regular expressions.

### Scientific documents collection

This study enabled a large automatic collection of published literature by accessing APIs designed by publishers for data mining purposes. The RSC and Elsevier provide full text of published articles. In order to collect these articles for data extraction, literature collection was carried out by combining Python HTTP libraries “urllib3” and “requests” with “Sodium ion battery” as the keyword and the search year was 2010 to 2023. The data returned by the server consisted of CSS, JavaScript, and image format documents, as well as HTML and XML files, which contained the complete structured content of each article required for this study. Accordingly, this study preliminary from Elsevier developer portal website (https://dev.elsevier.com/) obtained the 48750 articles, from the RSC (https://www.rsc.org/) to download the 14697 articles. A total of 63447 articles were initially stored in HTML/XML format.

### Data post-processing

To combine the relation extraction records from the synthesis parameter and electrochemical property and create uniform records for a formatted database. the following data post-processing is adopted:

1. Sintering temperature and time extracted are used as synthesis parameters for all materials under uniform Doi.
2. The voltage range is split into two properties: Voltage lower limit and voltage upper limit.
3. Under certain conditions, records of cycling performance can be used to calculate the capacity at a specific number of cycles by using the following formula:

1 $$Capacity\,retention\,{rate}_{{{\rm{C}}}_{i}}=\frac{{Capacity}_{{C}_{i}}}{{Capacity}_{{C}_{Initial}}}$$

Here, C_i_ denotes the number of cycles i, ${Capacity}_{{C}_{i}}$ represents the capacity under the ith cycle, ${Capacity}_{{C}_{Initial}}$ is usually defaulted to the first cycle discharge capacity of the battery.

**Fig. 12:**
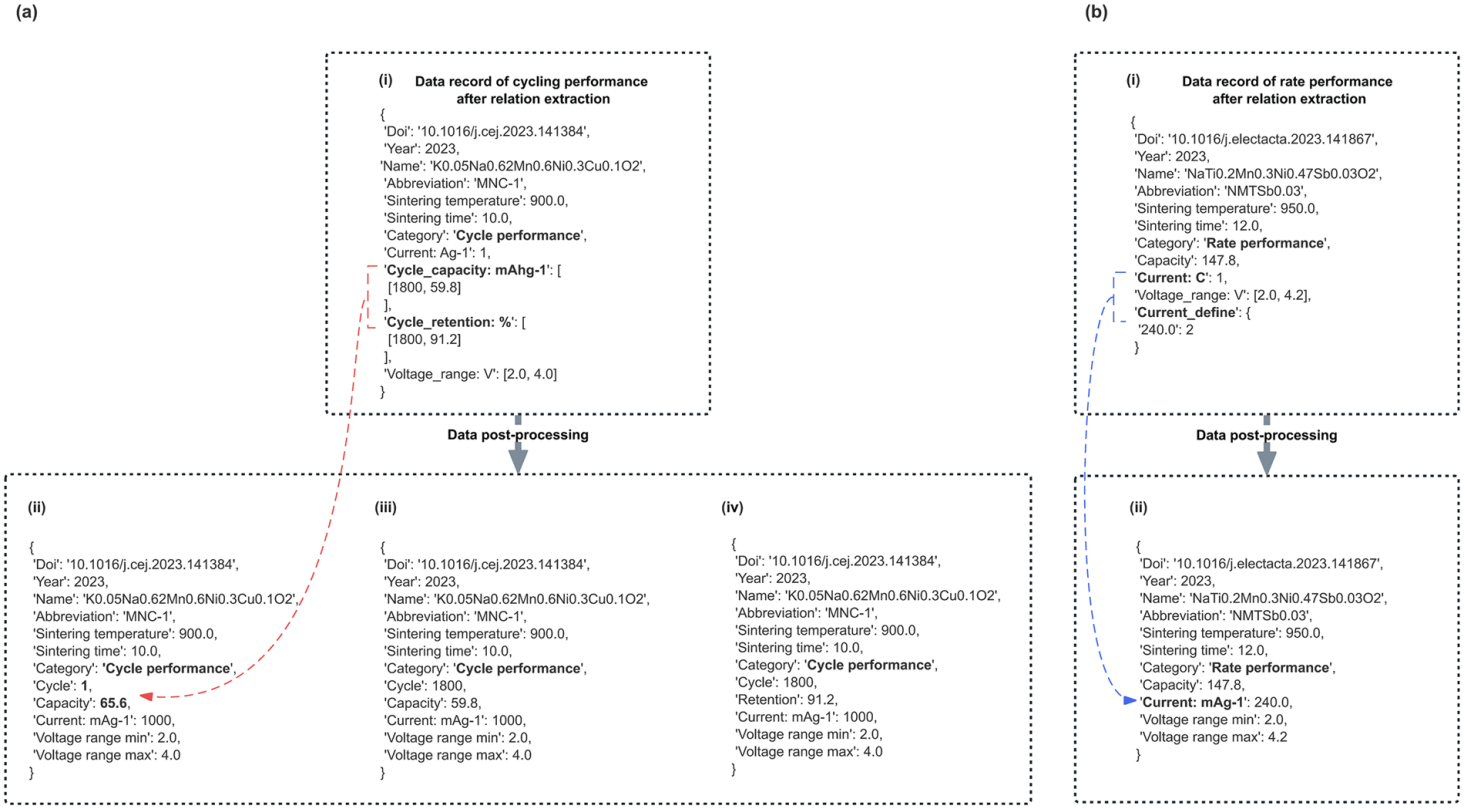
Data post-processing.

If this record contains both <the number of Cycles (referred to as C_i_), Capacity> and <the number of (referred to as C_j_), Capacity Retention> and if i = j, the initial capacity can be deduced. Additionally, if i = 1, the capacity under the number of C_j_ cycles can also be deduced. As depicted in Fig. 12a, (i) represents the initial data record, including “Cycle_capacity: mAhg-1” and “Cycle_retention: %”. The red line depicts the relationship derived from the data. By performing the calculation with i = j = 1800, $Capacity\,retention\,{rate}_{{{\rm{c}}}_{1800}}=91.2 \%$ and ${Capacity}_{{C}_{1800}}=59.8\left(mAh\,g-1\right)$, the initial capacity is determined to be 65.6 (mAh g-1). The post-processed data record is represented as Fig. 12a, (ii).

Supplementary Tables units of C and Ag-1 have been converted to mAg-1 for consistency. The conversion factor for 1 C-rate (mA g-1) is determined using the “Current definition” rule in Supplementary Table [1]. The highest frequency of the “Current definition” records is selected to establish the conversion factor. Fig. 12b, illustrates the mapping relationship between 1 C-rate (mA g-1) and the corresponding frequency mentioned in the article. The blue line represents the flow of the current unit conversion from “C” to “mAg-1”. By standardizing the current density units to “mAg-1”, the data can be presented consistently throughout the article. This conversion ensures clarity and facilitates accurate comparison and analysis of the results.

These data post-processing steps ensure that the extracted data records are in a consistent format and can be easily integrated into a formatted database.

## Technical Validation

To assess the effectiveness of the methods utilized in this study, precision, recall, and F1 score were employed as evaluation metrics for various subtasks, including abstract, paragraph, and sentence classification, chemical abbreviation definition identification, and relation extraction. Both precision and recall have a probabilistic interpretation. Precision measures the probability of the system correctly identifying a relevant object, while recall measures the probability of a relevant object being correctly identified^54^. Precision is calculated by quantifying the number of positive class predictions that truly belong to the positive class, while recall quantifies the number of positive class predictions made from all positive examples in the dataset. The F1 score, on the other hand, provides a balanced mean of precision and recall, considering their relationship.

2 $${Precision}_{c}=\frac{{TP}_{c}}{{TP}_{c}+{FP}_{c}}$$

3 $${Recall}_{c}=\frac{{TP}_{c}}{{TP}_{c}+{FN}_{c}}$$

4 $$F1\,{score}_{c}=\frac{2\ast {Precision}_{c}\ast {Recall}_{c}}{{Precision}_{c}+{Recall}_{c}}$$

where TP represents true positives, FP represents false positives, FN represents false negatives, and binary evaluations are performed for each label “c”.

According to Tsoumakas *et al*.^55^, micro-F1 and macro-F1 are versions of the F1 score that are micro-averaged and macro-averaged, respectively. These metrics are used to evaluate the combined performance of binary classification predictions for multi-labels. Therefore, in order to comprehensively evaluate the multi-label classification task for the abstract, this study considers precision, recall, and F1 score for each category, as well as their corresponding micro-averaged and macro-averaged versions. The formulas for these metrics are as follows:

5 $${Precision}_{micro}=\frac{{\sum }_{c=0}^{n}{TP}_{c}}{{\sum }_{c=0}^{n}{TP}_{c}+{\sum }_{0}^{n}{FP}_{c}}$$

6 $${Recall}_{micro}=\frac{{\sum }_{c=0}^{n}{TP}_{c}}{{\sum }_{c=0}^{n}{TP}_{c}+{\sum }_{c=0}^{n}{FN}_{c}}$$

7 $$F1\,{score}_{micro}=\frac{2\ast {Precision}_{micro}\ast {Recall}_{micro}}{{Precision}_{micro}+{Recall}_{micro}}$$

8 $${Precision}_{macro}=\frac{{\sum }_{c=0}^{n}{Precision}_{c}}{n}$$

9 $${Recall}_{macro}=\frac{{\sum }_{c=0}^{n}{Recall}_{c}}{n}$$

10 $$F1\,scor{e}_{macro}=\frac{{\sum }_{c=0}^{n}F1\,scor{e}_{c}}{n}$$

### Supplementary information

Supplementary Table [1]

## Data Availability

The dataset extracted by our pipeline has been made available in CSV and JSON formats at figshare^56^. The details of the data format are given in Table 7.

## Code availability

The source code of the document-level information extraction pipeline is available at https://github.com/GGNoWayBack/cathodedataextractor.
