# Supplementary material for: A document-level information extraction pipeline for layered cathode materials for sodium-ion batteries
Source: Sci Data. 2024 Apr 11;11:372. doi: 10.1038/s41597-024-03196-1 (PMC11009284; doi:10.1038/s41597-024-03196-1)
Supplement: Supplementary file 1 — Supplementary Table [1] [file 41597_2024_3196_MOESM1_ESM.docx]

Table S1: Patterns, examples, and rules for property value and unit in the field of battery research

| **Paragraph categories** | **Property name** | **Examples** | **Matching rules (Regular expression)** |
| --- | --- | --- | --- |
| *Experiment* | *Temperature* | 1400, 1500, and 1550°C | \d{2,3}+[-/(\d, airtond)]*°C(?![\s/]*min[-1]*) |
|  | *Time* | 12 hours, 12h | \b\d[-/(\d,. orand)]*h(?>rs\|oursh\|ours?)?\b(?! to reach) |
| *other than Introduction* | *Cycle* | 100 cycles | (?(DEFINE)(?P<FIRST>(initial\|first))  (?P<CHARGE>(charge\|discharge)\W(?3)*))  (?<= )\b(\d++[\d,/ andoghscueirTtn]*? (?=cycle\|(?&CHARGE) capacit))  \|(?&FIRST)(  \|( (?&CHARGE))?( specific\| reversible)? capacit(?>y\|ies)(?=.+?mAhg-1)  \|[ -]*((?&CHARGE)\|cycle)) |
|  |  | first capacity |  |
|  |  | initial discharge capacity |  |
|  | *Capacity* | 117.3, 120.3, and 110.1 mAhg-1 | (?<![-=])\b\d[\d.,~/ toand]*+mAhg-1 |
|  | *Retention* | capacity retention of 55% | (?<=\bretention.)[^%]*+%(?! higher)([^%](?>[^%]+\|(?1))%(?! higher))*  \|(?<=[ ~()])\d[\d.,and ]*+%[^%]+? (?=capacity\|retention) |
|  | *Voltage range* | 2.0-4.8V,  2.0 and 4.5V | (\d[\d.]*+)\s?(?>-\|to\|and)\s?(\d[\d.]*+)\s?(?=V\b) |
|  | *Current* | 5, 10 and 20 C | (?<![-.])\b(\d[\d.,toand ]*+C\b(?!=)\|(?<fraction>(?<![°@])C\s?/\s?\d+)) |
|  |  | 75 and 300 mAg-1 | (?<![-=])\b\d[\d.,~/ toand]*+mAg-1 |
|  |  | 0.1, 0.4 and 1.0Ag-1 | (?<!-)\b\d[\d.,/ toand]*+Ag-1 |
|  | *Current definition* | 1C=150mAg-1 | (?(DEFINE)(?P<NUMBER>(initial\|first))  (\b(?&NUMBER)C\s?=\s?(?& NUMBER)\s?mAg-1\b\|  (?&NUMBER)\s?\ ((?&NUMBER)C\s?\)\|  (?&NUMBER)C\s?\((?& NUMBER)\s?mAg-1\s?\)) |
|  |  | 20mAg-1 (0.1 C) |  |
|  |  | 0.2C (50mAg-1) |  |
